# Infection control in ERCP using a duodenoscope with a disposable cap (ICECAP): rationale for and design of a randomized controlled trial

**DOI:** 10.1186/s12876-020-01200-7

**Published:** 2020-03-12

**Authors:** Nauzer Forbes, B. Joseph Elmunzer, Thibault Allain, Millie Chau, Hannah F. Koury, Sydney Bass, Paul J. Belletrutti, Martin J. Cole, Emmanuel Gonzalez-Moreno, Ahmed Kayal, Puja Kumar, Rachid Mohamed, Christian Turbide, Andre G. Buret, Steven J. Heitman

**Affiliations:** 1 Division of Gastroenterology and Hepatology, Department of Medicine, University of Calgary, Calgary, Canada; 2 Department of Community Health Sciences, University of Calgary, Calgary, Canada; 3 Division of Gastroenterology and Hepatology, Medical University of South Carolina, Charleston, SC USA; 4 Department of Biological Sciences, University of Calgary, Calgary, AB Canada; 5 Cumming School of Medicine, University of Calgary, Calgary, AB Canada

**Keywords:** ERCP, Duodenoscopes, Infection control, Sepsis

## Abstract

**Background:**

Endoscopic retrograde cholangio-pancreatography (ERCP) is commonly performed in the management of pancreatic and biliary disease. Duodenoscopes are specialized endoscopes used to perform ERCP, and inherent to their design, a high rate of persistent bacterial contamination exists even after automated reprocessing and disinfection. Consequently, in recent years, ERCP has been associated with infection transmission, leading to several fatal patient outbreaks. Due to increasing fears over widespread future duodenoscope-related outbreaks, regulatory bodies have called for alterations in the design of duodenoscopes. A duodenoscope has recently been developed that employs a disposable cap. This novel design theoretically eliminates the mechanism behind persistent bacterial contamination and infection transmission. However, there are no data demonstrating persistent bacterial contamination rates, technical success rates, or clinical outcomes associated with these duodenoscopes.

**Methods:**

A parallel arm randomized controlled trial will be performed for which 520 patients will be recruited. The study population will consist of consecutive patients undergoing ERCP procedures for any indication at a high-volume tertiary care centre in Calgary, Alberta, Canada. Patients will be randomized to an intervention group, that will undergo ERCP with a novel duodenoscope with disposable cap, or to a control group who will undergo ERCP with a traditional duodenoscope. Co-primary outcomes will include persistent bacterial contamination rates (post automated reprocessing) and ERCP technical success rates. Secondary outcomes include clinical success rates, overall and specific early and late adverse event rates, 30-day mortality and healthcare utilization rates, procedure and reprocessing times, and ease of device use.

**Discussion:**

The ICECAP trial will answer important questions regarding the use of a novel duodenoscope with disposable cap. Specifically, persistent bacterial contamination, technical performance, and relevant clinical outcomes will be assessed. Given the mortality and morbidity burden associated with ERCP-related infectious outbreaks, the results of this study have the capacity to be impactful at an international level.

**Trial registration:**

This trial was registered on clinicaltrials.gov (NCT04040504) on July 31, 2019.

## Background

Endoscopic retrograde cholangiopancreatography (ERCP) is a well established modality used in the management of benign and malignant pancreatico-biliary pathology [1–4]. While effective and safe overall, the performance of ERCP is associated with several recognized adverse events, including post-ERCP pancreatitis (PEP), bleeding, cholangitis, cholecystitis, perforation, and cardiopulmonary events [5].

ERCP is performed using a duodenoscope, whose unique design affords direct visualization of the major and/or minor duodenal papillae, necessary for successful biliary and/or pancreatic cannulation and instrumentation. A recess in the tip of the duodenoscope houses the elevator, a device designed to assist with fine tip control of pancreatico-biliary cannulation and extraction devices. Due to this design, duodenoscopes are among the most complicated medical devices to disinfect and reprocess. This is mainly a result of the largely inaccessible elevator recess, which makes manual or automated cleansing and disinfection difficult, and therefore promotes persistent pathogenic colonization of the duodenoscope. Up until the early 1990s, ERCP-related infection transmission, while occurring rarely, had always been associated with breaches in published cleaning, disinfecting or drying protocols [6].

Recently, a sharp rise in the rate of duodenoscope-related infections has come to the forefront. At least 25 distinct outbreaks caused by contaminated duodenoscopes have been identified worldwide between 2012 and 2015 alone, affecting over 250 patients [7–9]. Surprisingly, the majority of these outbreaks have been unrelated to any identifiable breaches in disinfection and reprocessing protocols, and there has been no apparent association with geographic location, duodenoscope manufacturer or model [7]. This has called into question the robustness of reprocessing processes as well as traditional duodenoscope designs. More worrisome is the involvement of multidrug-resistant organisms (MDROs), which portend significantly higher rates of patient morbidity and mortality compared with traditional organisms. Carbapenem-resistant Enterobacteriaceae (CRE), a family of gram-negative bacteria repeatedly linked to duodenoscope-related infections, is resistant to almost all classes of available antibiotics [9–11]. A 2018 nationwide European study demonstrated that nearly 40% of sites providing ERCP had one or more patient-ready duodenoscope(s) that were contaminated with common bacteria after standard processing [12], suggesting that our current disinfection protocols are inadequate. There have been increases in the rates of MDRO transmission over the past decade [13]. The mortality rates associated with duodenoscope-related MDRO outbreaks can be extremely high, with a 1-month mortality rate of over 20% reported in one American study [14].

Novel strategies including refined endoscope designs are necessary in order to prevent future ERCP-related outbreaks. Existing sterilization and processing protocols are inadequate given the increased resistance of the microbes colonizing duodenoscopes, particularly behind the inaccessible elevator recess. Multiple manufacturers are therefore attempting to alter the design of traditional duodenoscopes to address this vital issue. Disposable duodenoscopes are currently being developed to circumvent the sterilization process entirely, but the projected costs associated with such devices may ultimately make them unfeasible for use at medium- to high-volume centers [15].

Alternatively, specialized duodenoscopes with disposable caps have been developed with the elevator mechanism housed entirely within the disposable piece. This theoretically eliminates the primary mechanism behind persistent duodenoscope colonization and patient transmission. Therefore, these devices are a promising new modality that have the potential to drastically reduce or eliminate the rates of duodenoscope-related infection, if widely adopted. However, their clinical efficacy and residual contamination rates remain unproven, and patient outcomes following their use are unknown.

## Methods

### Study design and setting

This protocol (version 7.0) was written and reported according to the Standard Protocol Items: Recommendations for Interventional Trials (SPIRIT) recommendations [16, 17], and a SPIRIT checklist is provided in Supplemental 1. This is a consecutive parallel arm phase IV RCT which will assess 1) the persistent bacterial contamination rate (PBC) of a duodenoscope with a disposable elevator cap (DEC) versus a traditional duodenoscope and 2) the technical and clinical efficacy of the DEC duodenoscope versus a traditional duodenoscope. The study will take place at a single high-volume tertiary ERCP referral center in Calgary, Alberta, Canada, where over 1500 ERCPs are performed annually. Here, ERCP-related care is provided to patients of Calgary (population 1.4 million) and surrounding areas, comprising a total catchment population of close to 2 million. All procedures will be performed by experienced endoscopists having each performed over 1000 ERCPs or by advanced therapeutic endoscopy trainees under direct supervision of the expert endoscopists.

### Study population

All consecutive patients at the study center who meet the eligibility criteria below will be invited to participate in the study. For cases in which it is determined that the patient failed to meet the eligibility criteria after the patient has agreed to participate in the study and/or has signed the written informed consent form, the study personnel will complete screening forms as well as indicate the specific inclusion/exclusion criterion that was not met. Such a patient will be considered a screen failure and will not be included the final analyses.

### Device description

**Fig. 1:**
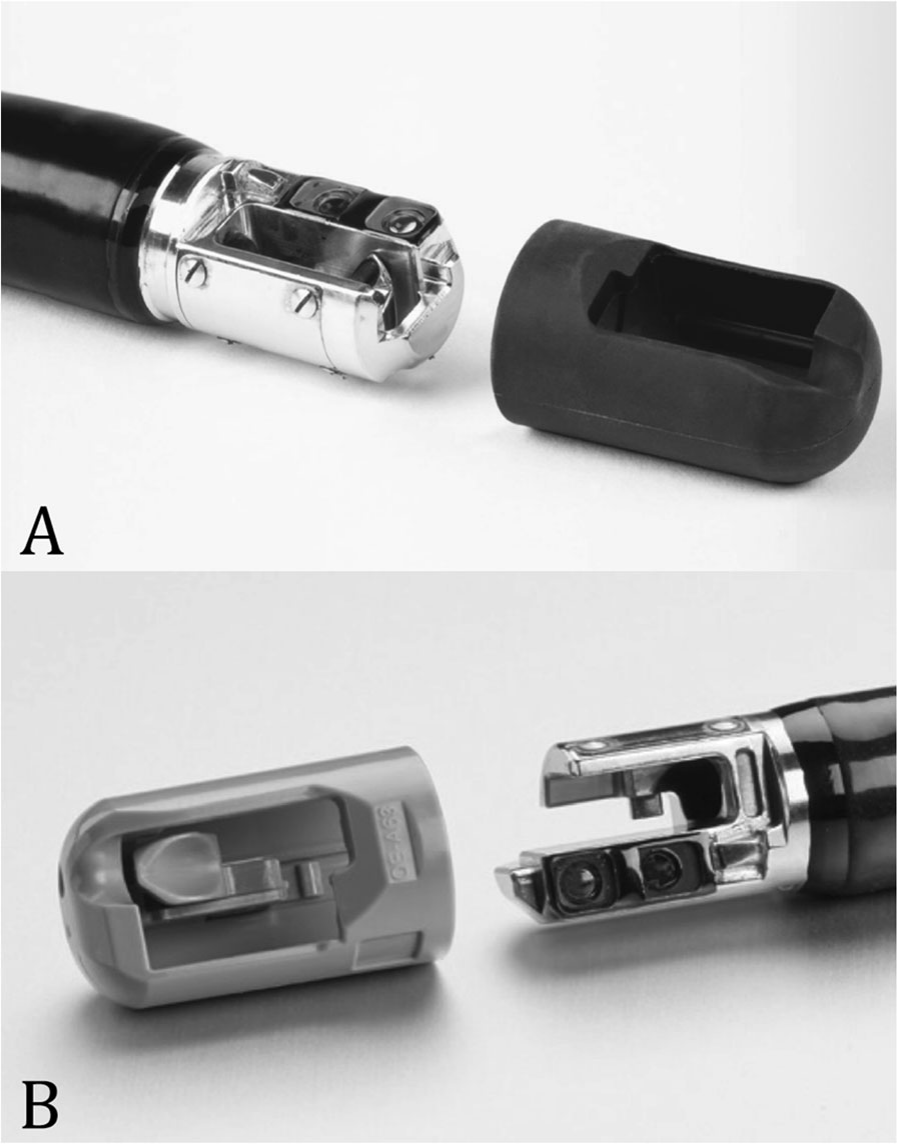
**a** ED34-i10T duodenoscope and **b** ED34-i10T2 duodenoscope with DEC™ [18, 19]

This study will be conducted using approved commercially available devices. All devices will be used in accordance with the appropriate Directions for Use (DFU). The Pentax ED34-i10T2 duodenoscope is specifically designed for use with the DEC™ disposable elevator cap. It offers high-definition endoscopic visualization and ergonomic handling while affording the opportunity for complete removal and disposal of the duodenoscope cap, which houses the elevator mechanism. The Pentax ED34-i10T model is a standard high-definition duodenoscope currently used at our centre and will serve as the control instrument. Though it has a detachable tip for ease of manual distal end access, the elevator mechanism remains a part of the scope, and the tip is non-disposable, needing to be reattached to the rest of the scope after cleaning. The interventional and control instruments are shown in Fig. 1. All duodenoscopes used for this study will be less than 24 months old, and their daily use will be comparable. All scopes will undergo scheduled routine maintenance and will be removed from study or clinical use if any damage is known, only being returned once repaired. These steps will be taken acknowledging the association between duodenoscope damage and residual contamination [20]. There are 4 DEC T2 duodenoscopes that will be used throughout this study and 6 standard T1 duodenoscopes. All duodenoscopes will be reprocessed by the same automated endoscope reprocessor and stored in a common cabinet after reprocessing.

### Eligibility criteria

Patients will be required to meet *all* of the following inclusion criteria in order to be eligible for study participation:

- age ≥ 18 years;
- ability to give written informed consent to involvement;
- requirement for ERCP to be performed for any indication.

Patients meeting *any* of the following exclusion criteria will not be eligible for study participation:

- standard contraindications to ERCP (e.g. hemodynamic instability or uncontrolled coagulopathy);
- pregnant or breastfeeding status;
- inability to successfully complete an ERCP procedure under conscious sedation;
- out-of-province status (which could prohibit follow-up);
- incarceration (which could prohibit follow-up).

### Randomization, allocation and crossover

Patients fulfilling the eligibility criteria above will be randomized following written consent in a 1:1 ratio. Permuted block randomization will be utilized in blocks of 8 patients. Allocation will be to one of two groups: 1) ERCP using ED34-i10T2 duodenoscope with DEC or 2) ERCP using a traditional duodenoscope (ED34-i10T). Confidential random number allocation will be accessed via the secure internet-based randomization program *randomize.net*. Outcome adjudicators, patients, and those who conduct and interpret the analyses will be blinded to allocation. The endoscopist will not be blinded. The decision to cross over from the disposable-cap duodenoscope to the standard duodenoscope in order to achieve the desired goal of the procedure will be left to the discretion of the treating endoscopist, but will be considered a technical failure of the ED34-i10T2/DEC.

### Study sequences

Consecutive patients undergoing ERCP will be flagged as potentially eligible for study participation. On the day of the procedure, the patient will be approached by a research assistant (RA), who will explain the study, confirm eligibility, and answer any potential questions. If the patient agrees to participate, the relevant written informed consent form is signed and witnessed. If the patient decides not to participate, the ERCP proceeds as per the usual standard of care. Duodenoscopes are stored in a cabinet in the ERCP unit and will be clearly marked and color-coded according to study group assignment.

After the ERCP is complete, the in-room nursing team will carry out standardized post-endoscopy manual cleaning and disinfection of the DEC or traditional duodenoscope. In cases where the DEC was used, the cap will be disposed of. These processes will be timed by the research assistant. Following this, the scope will be portered to the automated reprocessing room for disinfection and reprocessing, which will also be timed.

After reprocessing, staff will then observe the following sequence to determine whether there is any PBC of the duodenoscope after standardized disinfection and reprocessing. These samples will then be shipped for microbiologic analysis. Our unit’s protocol currently dictates that reprocessed scopes undergo point-of-care bioluminescence scans to rule out persistent contamination. Scopes that fail bioluminescence testing are required to be reprocessed anew until the bioluminescence scan is negative. This will take place routinely independent of the study; however, the only change for study scopes will be that our study’s sampling protocol (described below) will take place regardless of the bioluminescence result.

The protocol for duodenoscope sampling that will be utilized for this study is adapted from the Duodenoscope Surveillance Sampling and Culturing Protocols, which were developed jointly by the Department of Health and Human Services (DHHS), Food and Drug Administration (FDA), Centers for Disease Control and Prevention (CDC), and American Society for Microbiology (ASM) [21]. This procedure is summarized here, and will occur within 1 h of the completion of reprocessing.

1. Two staff are needed to conduct sampling from duodenoscope channels; one person (the sampler) will maintain aseptic handling and conduct brushing steps, while the second person (the facilitator) will open packages and handle the unsampled portions of the duodenoscope;
2. The staff will label the sterile sample containers with relevant information;
3. The staff will don their personal protective equipment (PPE);
4. The staff will set the duodenoscope on a sterile drape;
5. Two samples will be collected and combined. First, an instrument channel sample (from the biopsy port to the distal scope end) will be taken;
6. Second, an elevator recess sample will be taken by brushing and flushing of the elevator recess. In the case of the disposable cap, the distal scope tip to which the disposable cap attaches will be sampled instead, ensuring sampling of the joint line between cap and scope.
7. The duodenoscope will then be handled and transported back to the endoscopy unit according to our local institutional policies;
8. Samples will be packed in appropriate biohazard containers, packaged, and transported for analysis to our microbiology laboratory, located within 30 min driving distance of the hospital. The samples will be assigned a unique study number so that the microbiologic analysts will be blinded to the type of duodenoscope that was used for the index procedure.

The presence of any microbial population(s) will be assessed using plating for growth in aerobic conditions. 0.1 mL of wash fluid will be plated to blood agar, MacConkey agar, and Columbia blood agar with colistin-nalidixic acid. If after 72 h there is no growth, the sample will be deemed negative, and will be discarded. Positive samples will be further characterized. Growth will be reported in colony-forming units (CFU)/mL. For Gram-negative bacilli (GNB), these will be reported with CFU/mL and the isolated organism(s) will be reported as identified by matrix-assisted laser desorption/ionization (MALDI). Non-GNB organisms will be reported only if CFU/mL is greater than 20, and a group identification (ie, coagulase-negative *Staphylococcus*, or *Candida*) will be provided.

**Fig. 2:**
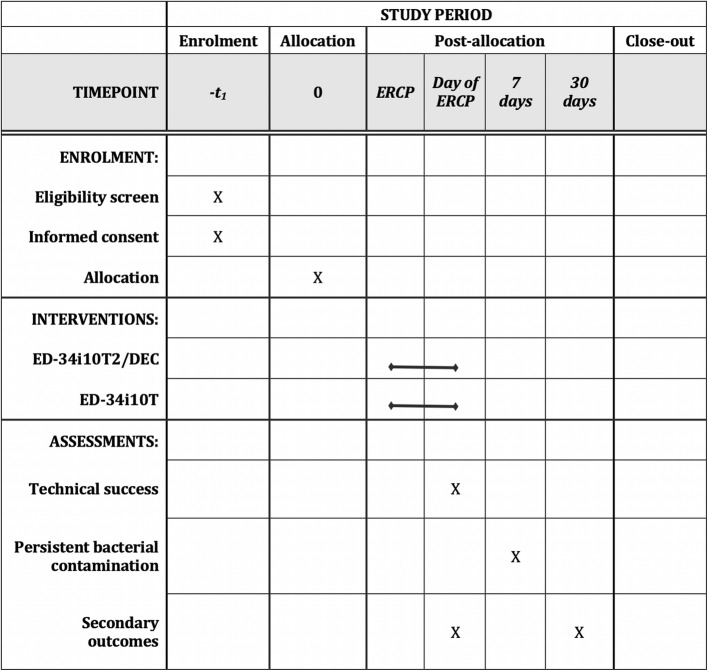
SPIRIT schedule of enrolment, interventions and assessments [16, 17]

Patients will be contacted by the RA by telephone 30 days following their ERCP to assess for any ongoing symptoms and advise of any adverse events, including unplanned emergency department visits or inpatient admissions. The patient’s medical record is also reviewed at 30 days for complete details of any unplanned emergency department visits, inpatient admissions or prolonged admissions. In accordance with the Declaration of Helsinki and the International Conference on Harmonisation of Technical Requirements for Registration of Pharmaceuticals for Human Use Good Practice Guidelines, a participant is free to withdraw from participation in the study at any time, for any reason without prejudice to their future medical care by the physician or the institution. Investigators can also withdraw patients from the study at any time due to potential concerns over safety. Unblinding can occur at this stage if necessary. Data collected up to the time of withdrawal will be analyzed. Study sequences are summarized in Fig. 2.

### Study outcomes

There are two co-primary outcomes for this study. The first primary outcome is the PBC rate with the ED34-i10T2 duodenoscope and DEC, compared to the currently used ED34-i10T duodenoscope, following standardized disinfection and reprocessing. PBC will be defined as growth of any identifiable microorganism at ≥20 colony forming units (CFU) per 20 mL of solution [22].

The second primary outcome of the study is the ERCP technical success rate with ED34-i10T2/DEC, compared to ED34-i10T. Technical success will be determined in duplicate by two blinded outcome adjudicators with no knowledge of the patient’s study group allocation. The adjudicators will review redacted and de-identified procedure reports for each study patient, and determine the presence or absence of technical success of the overall procedure based on a set of a priori definitions (Tables 1 and 2). Redacted elements will include type of duodenoscope used, specific endoscopists or trainees performing the procedure, and any identifiable patient information including name, date of birth, and health record numbers. Disagreements will be resolved by a third blinded adjudicator. Technical success will also be determined subjectively on a case-by-case basis by the endoscopist performing the procedure, in order to measure correlation with the adjudicator determinations as a secondary analysis, but will not be used in the determination of outcome for the primary analysis. Failure of the procedure due to inability to safely perform the ERCP under conscious sedation will not constitute a technical failure for purposes of this study, in either study arm.

**Table 1:** Definitions of technical success by ERCP indication

Indication for ERCP	Definition(s) of Technical Success
Suspected or confirmed bile duct stone(s)	Extraction of stone(s) OR CBD clearance based on absence of filling defects on occlusion cholangiogram [If difficult biliary stones are encountered during procedure, use ‘Difficult biliary stone(s)’ indication below, and above definition does not apply]
Difficult biliary stone(s) [23–25] – any of: • One or more stone(s) ≥ 15 mm • Barrel or other unusual shape • Multiple (4 or more stones) • Impacted stone(s) • Intrahepatic or cystic duct stone(s) • Stricture below stone(s)	Extraction of stone(s) OR CBD clearance based on absence of filling defects on occlusion cholangiogram OR Stenting of CBD as part of future plan to clear duct
Biliary stricture (benign or malignant)	Successful placement of stent with proximal margin proximal to stricture OR Successful dilatation of stricture
Cholangioscopy or pancreatoscopy	Successful cholangioscopic or pancreatoscopic visualization of area of interest
Chronic pancreatitis, pancreatic stone(s) and/or pancreatic stricture(s)	Successful cannulation of main pancreatic duct (PD) AND AT LEAST 1 OF: Pancreatic sphincterotomy Stenting or dilatation of PD Extraction of PD stone(s)
Pancreas divisum	Successful minor papilla cannulation AND Successful pancreatic sphincterotomy
Stent removal or exchange	Successful removal and/or exchange of stent(s)
Treatment of peri-ampullary bleeding	Successful endoscopic hemostasis
Sphincter of Oddi dysfunction	Successful biliary sphincterotomy

**Table 2:** Reasons for technical failure by ERCP indication

Reason(s) for Technical Failure	
Inability to locate papilla in patient with normal anatomy	
Inability to locate papilla in patient with altered anatomy, including: • duodenal or peri-ampullary diverticulum • Billroth II surgery • Roux-en-Y surgery	
Inability to achieve an en-face view of the papilla of interest due to a luminal stricture	
Inability to cannulate duct of interest	
Inability to perform sphincterotomy when necessary	
Inability to clear duct of interest	
Inability to place stent proximal to area of interest	
Inability to remove or exchange stent	
Inability to achieve hemostasis endoscopically	
Inability to successfully load, exchange or remove devices necessary for the completion of the procedure Inability to safely complete procedure due to issues with sedation^a^	

^a^If this is the only reason for technical failure, the patient will be excluded from final analysis

Secondary outcomes include clinical success rates, overall and specific early (intraprocedural) and late (30-day) adverse event rates, 30-day mortality and healthcare utilization rates, procedure and reprocessing times, and ease of device use. Secondary study outcomes and their definitions are listed in Table 3.

**Table 3:** Secondary study outcomes

Secondary Outcome	Definition
Clinical success rate	Technical success, in addition to a lack of repeat unplanned endoscopy, imaging, emergency department presentation or admission within 30 days of the index procedure for reasons related to ongoing pancreatico-biliary pathology that was initially thought to be resolved after the index ERCP
Subjective presence technical success	Determined by endoscopist, in binary fashion
Subjective assessment of duodenoscope ease of use	Determined by endoscopist, using Likert scale of 1–10
Dislodgement rate of the disposable cap (for interventional arm only)	Loss of the duodenoscope cap inside the patient
Overall adverse event rate	Any adverse event(s) occurring within 30 days of the index procedure; divided into intraprocedural, early and late in terms of timing, and characterized in terms of severity by the ASGE Lexicon
Pancreatitis rate	New or distinct abdominal pain after ERCP in addition to lipase rise above 3 times the upper limit of normal, within 30 days of the index procedure
Asymptomatic lipase rate	Lipase rise above 3 times the upper limit of normal, within 30 days of the index procedure, not accompanied by classic pancreatitis abdominal pain
Bleeding rate	Hematemesis and/or melena and/or hematochezia, or drop in hemoglobin by ≥2 g following ERCP with either sphincterotomy or sphincteroplasty (or both) within 30 days of the index procedure
Cholangitis and/or sepsis rate	Re-presentation, readmission or prolongation of admission for any suspicion of biliary sepsis, indicated by any of: positive bacterial blood culture(s), leukocytosis, fever, or other features of systemic inflammatory response syndrome (SIRS), within 30 days of the index procedure
Unplanned presentation rate	Hospital admission or unplanned presentation to an acute healthcare facility within 30 days of the index procedure
Mortality rate	Patient death within 30 days of the index procedure
Manual post-ERCP disinfection time	Time taken to manually swab and disinfect the duodenoscope in the endoscopy unit after completion of ERCP
Automated post-ERCP reprocessing time	Time taken to automatically reprocess the duodenoscope in the reprocessing department after completion of ERCP

### Data management

The investigators have previously designed and implemented the Calgary Registry for Advanced and Therapeutic Endoscopy, CReATE [26]. CReATE is a high-fidelity prospective database that collects over 300 data fields in real-time for each ERCP procedure, including detailed patient, endoscopist, procedure and post-procedure variables. Therefore, CReATE serves as the ideal data collection platform for this randomized trial. Data are inputted by a full-time research assistant, and stored and managed on a secure encrypted web application specializing in confidential healthcare database implementation and maintenance [27].

De-identified study variables (accessible only to study investigators) will include patient demographics, comorbidities, and relevant medications, endoscopist procedural volume, presence and stage of trainees, degree of procedural involvement of trainees, ERCP indication, procedural details, procedure time, intraprocedural adverse event(s), and sedating medication(s).

### Statistical considerations

For primary outcome 1, a 2018 study [12] demonstrated a PBC rate of over 20% after standard duodenoscope disinfection, but with limited sample size for Pentax scopes. In addition, preliminary results from the FDA in 2019 in real-world settings demonstrated a 9.3% total sample positivity rate among Pentax duodenoscopes [28]. We thus propose that reduction in PBC from 10% with ED34-i10T to 3% with DEC (a relative risk reduction of 67%) would be considered clinically significant. Using a power of 80% with a two-sided alpha of 0.05, 194 subjects in each arm will be required, for a total of 388 patients. Four hundred twenty-six patients will be recruited, accounting for a 10% sample loss rate.

For primary outcome 2, we selected a baseline ERCP technical success rate of 92% based on our centre’s prospective data over the last 12 months. We propose that technical success of DEC within a margin of 7% will result in an ability to conclude non-inferiority based on the importance of avoiding additional MDRO outbreaks. Using a power of 80% with a one-sided alpha of 0.025 [29], 236 subjects in each arm will be required, for a total of 472 patients. Five hundred twenty total patients will be recruited, accounting for a 10% attrition rate. Primary outcome 2 and all secondary outcomes will be determined via both intention to treat (ITT) and per protocol analyses.

### Data monitoring committee

Data and safety monitoring will be performed by a committee of two independent study monitors. The monitors will ensure ongoing quality of data collection for the co-primary and secondary outcomes. The monitors will also review safety outcomes in both study arms and inform the investigators if there are any unforeseen trends. Any important safety outcomes or protocol amendments will be reported on clinicaltrials.gov and to our local institutional ethics board.

### Study integrity, ethics and registration

No industry-related funding has been received to support this study or to compensate study investigators. The temporary use of the DEC duodenoscopes has been granted by Pentax Canada. Our study has received full ethics approval from the Conjoint Health Research Ethics Board (CHREB) at the University of Calgary (REB 19–0983). Our study is registered on clinicaltrials.gov (NCT04040504). There are no planned audits into trial conduct given there is no funding from industry sponsors. Dissemination of study results is planned through publication at medical conferences and/or in peer-reviewed journals.

## Discussion

This RCT will be the first study to assess the clinical performance and persistent bacterial contamination rates of a novel duodenoscope with disposable cap, compared to a currently used duodenoscope model. ERCP is a common procedure, with over 450,000 cases performed annually in the United States alone [30]. ERCP-related outbreaks are highly feared outcomes for patients, practitioners and institutions alike. There has been considerable world-wide media coverage of this important patient safety issue in recent months. This has ultimately led the FDA to release guidance actively encouraging the design and use of novel duodenoscopes that facilitate or eliminate the requirement for reprocessing [31]. Furthermore, several endoscopic societies have made it a priority to address duodenoscope designs and reprocessing procedures [22, 32].

Recruitment for this study is planned to start in late fall of 2019 and expected to conclude by the end of 2020, given our centre’s high procedural volumes. Loss to follow-up is expected to be low, given that the co-primary outcomes are determined at or shortly after randomization and given that we have ethics approval to formally review patients’ provincial health records at the 30 day mark. This, in combination with a planned routine phone call from a research assistant, ensures the capture of presentations to any health facility for reasons related to the index procedure. The ED34-i10T2/ DEC duodenoscope has already been approved for clinical use in Canada, and preliminary trials at our centre are encouraging, leading us to hypothesize there will be non-inferiority of this device compared with our currently used traditionally designed duodenoscope model. FDA approval is anticipated within 6 months, which will permit commercial availability in the United States. Though the DEC theoretically eliminates the mechanism behind ERCP residual contamination and infection transmission, this remains unproven. Moreover, data on technical performance and clinical outcomes are lacking. While formal cost effectivenesss analysis (CEA) will not be conducted as part of this study, important data on device effectiveness, clinical success, repeat healthcare utilization, procedure time, and reprocessing time will be acquired that can inform future CEAs.

In conclusion, this RCT will yield important answers regarding the persistent bacterial contamination rates, technical performance, and clinical outcomes of a novel duodenoscope making use of a disposable elevator cap. The results of our study could alter ERCP practices and outcomes in an immediate fashion. Given the optimal timing, the results of this study are potentially impactful on an international level.

## Supplementary information

**Additional file 1.** SPIRIT 2013 checklist: recommended items to address in a clinical trial protocol and related documents [16, 17].

## Data Availability

Not applicable. Once generated, data will be available through the corresponding author and through anticipated publication of study results.

## Abbreviations

ASGE: American Society for Gastrointestinal Endoscopy
ASM: American Society for Microbiology
CDC: Centers for Disease Control and Prevention
CEA: Cost effectiveness analysis
CFU: Colony forming unit
CHREB: Conjoint Health Research Ethics Board
CRE: Carbapenem-resistant Enterobacteriaceae
CReATE: Calgary Registry for Advanced and Therapeutic Endoscopy
DEC: Disposable elevator cap
DFU: Directions for use
DHHS: Department of Health and Human Services
ERCP: Endoscopic retrograde cholangiopancreatography
FDA: Food and Drug Administration
ITT: Intention to treat
MDRO: Multidrug-resistant organism
NGS: Next-generation sequencing
PBC: Persistent bacterial contamination
PPE: Personal protective equipment
RA: Research assistant
RCT: Randomized controlled trial
SIRS: Systemic inflammatory response syndrome
SPIRIT: Standard Protocol Items: Recommendations for Interventional Trials
